# Primary intraspinal dumbbell-shaped mesenchymal chondrosarcoma with massive calcifications: a case report and review of the literature

**DOI:** 10.1186/s12957-016-0963-9

**Published:** 2016-08-03

**Authors:** Shudong Chen, Yufeng Wang, Guoyi Su, Bolai Chen, Dingkun Lin

**Affiliations:** 1 Guangdong Provincial Hospital of Chinese Medicine, No. 111 Dade Road, Yuexiu District, Guangzhou, 510120 China; 2 Guangzhou University of Chinese Medicine, Guangzhou, China; 3 Guangdong Provincial Academy of Chinese Medical Sciences, Guangzhou, China

**Keywords:** Mesenchymal chondrosarcoma, Calcification, Spinal tumor, Surgery, Intraspinal

## Abstract

**Background:**

Mesenchymal chondrosarcoma is a rare malignant tumor arising from bone or soft tissues. Instraspinal dumbbell-shaped mesenchymal chondrosarcoma is even rarer; however, it should not be neglected by clinicians.

**Case presentation:**

A 26-year-old female was referred to our hospital with a 1.5-month history of sciatic pain and numbness in the left leg. Computed tomography and magnetic resonance imaging scans revealed an intraspinal dumbbell-shaped mass which had distinguishing features of neurogenic tumors, surprisingly, with massive calcifications, and no tumor metastasis was found. Then the patient underwent a total resection of the tumor, and during the operation, we found that the right nerve root of the fifth lumbar almost disappeared. The tumor was diagnosed as mesenchymal chondrosarcoma by histopathological examination after operation. Adjuvant therapies were not performed. However, recurrence of the tumor occurred 5 months later, and she underwent a total resection again combined with radiotherapy after second surgery.

**Conclusions:**

To the best of our knowledge, this case study presents the first report in literature about primary instraspinal dumbbell-shaped mesenchymal chondrosarcoma with massive calcifications, which may provide some evidence for clinical practice. As the clinical symptoms and radiographic findings of mesenchymal chondrosarcoma are usually not specific, clinicians should consider it as a possible case and diagnose it through careful histopathological examination. Sometimes, calcification could be seen in tumors, which may influence or reflect the growth of tumor and disease prognosis. Although prognosis in mesenchymal chondrosarcoma varies from person to person, generally, complete resection, adjuvant therapy, and regular examinations are recommended to perform for patients with mesenchymal chondrosarcoma.

## Background

Mesenchymal chondrosarcoma (MCS) is a malignant tumor arising from bone or soft tissues [1, 2], whose incidence accounts for 0.2–0.7 % of malignant bone tumors or 3–10 % of chondrosarcoma [3]. MCS most commonly originates in the bone, but it can also be found in extraskeletal sites, especially in the brain and meninges. However, intraspinal mesenchymal chondrosarcomas are rare, and the tumor with a dumbbell shape is even rarer. Sometimes solitary or punctate calcifications may occur in the primary MCS. To the best of our knowledge, there is no report about heavily calcified intraspinal dumbbell-shaped MCS. In the present study, we report a case of a dumbbell-shaped MCS with massive calcifications at the third to fifth lumbar in a 26-year-old female.

## Case presentation

A 26-year-old female patient suffering from sciatic pain and numbness in the left leg with a 1.5-month history was admitted to our department after a magnetic resonance imaging (MRI) scan which showed a tumor in her lumbar spinal canal. She had trouble in walking because of the pain, which can be relieved by maintaining on the right side with coxa and knee bending. Besides, she complained of urgency of urination and irregular bowel movement. Physical examination showed kyphosis of the lumbar spine, muscle atrophy of the left triceps surae, weakness of the anal sphincter, drop foot deformity on the right, hyporeflexia of the patellar tendon reflex on the left, absence of the Achilles tendon reflex, and the straight leg raising test, Yeoman test, and Ely test were positive. The muscle strength was 4/5 in left anterior tibial and extensor hallucis longus, while 0/5 in right. Signs and symptoms of drop foot, bladder, and bowel dysfunction were suspected to cauda equina syndrome, but she told us that she had the difficulty of dorsiflexion on the right foot since she was 14 years old, and aggravated gradually.

**Fig. 1:**
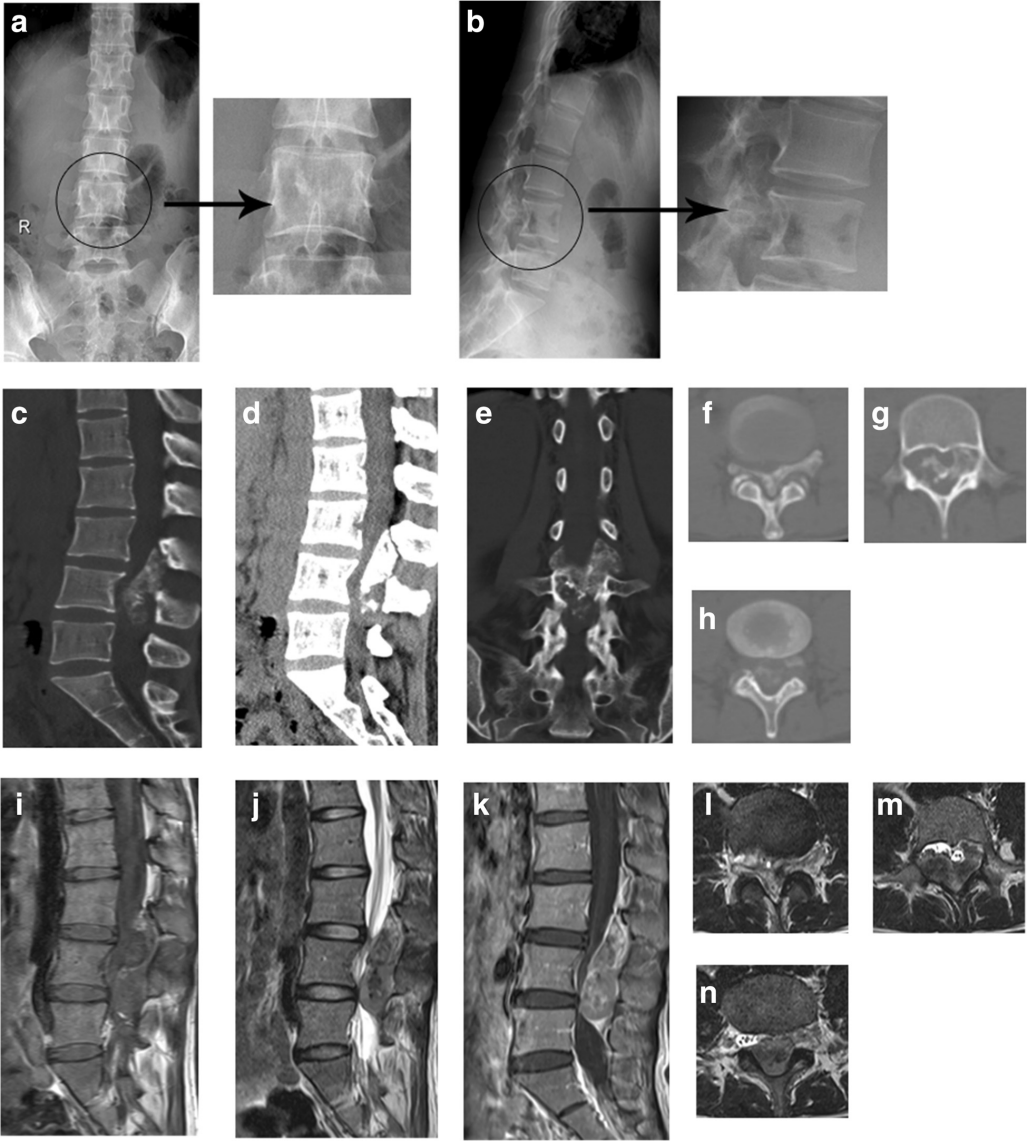
Images of X-rays and CT and MRI scans before operation. **a**, **b** Images of preoperative X-rays show unclear edge of left vertebral pedicle and posterior vertebra body of L4. **c**–**h** Preoperative CT images find massive calcifications within the mass. Images of **f**–**h** represent the level of L3, L4, and L5, respectively. **i**–**k** Images of preoperative MRI. Sagittal T1WI (**i**), T2WI (**j**), Gd-enhanced T1WI (**k**), and axial T2-WI (**l**–**n**) reveal a dumbbell tumor at the L3-5 level

Further checks were performed after admission. Results of laboratory tests including complete blood count, electrolytes, biochemical profile, erythrocyte sedimentation rate, and C-reactive protein were all normal. MRI revealed an intraspinal dumbbell-shaped mass measuring 1.8 × 2.3 × 6.2 cm with well-defined margin which extended from central canal to the intervertebral foramen. The tumor pushed forwards the dural sac, resulting in the cauda equine compressed and curved impression at the posterior of L4 vertebra. The mass showed isointense on T1WI images, low-signal intensity and mildly hyperintense mixed on T2WI images and fat-suppression T2WI, and heterogeneous density on the Gd-DTPA-enhanced T1WI. Computed tomography scans (CT) indicated that most parts of the tumor had calcified which extended towards both sides of intervertebral foramen at L3/4 and left side at L4/5, with intervertebral foramen enlarged, bone cortex thinned, and bone density of vertebral pedicle decreased (Fig. 1). According to these imagings, radiologist firstly thought the tumor to be type I neurofibromatosis.

Later, a L3-4 laminectomy was performed to remove the tumor. During the operation, we saw that the tumor was located intraspinal and adheres to the dura mater. It was red-and-gray, encapsulated, firm, well-circumscribed, and rich in vessels. More detailed examination revealed that the tumor had a haematoma in front of L4 lamina and mostly calcified at L3-4 level. Meanwhile, we could not find the right nerve root of L5 but only several nerve fiber tracts. It has also been confirmed in surgery that the right side of L4 vertebral pedicle had thinned, around which had hardened, thus making it very difficult to place a pedicle screw. Therefore, transpedicular screw fixation and facet joint bone autograft were performed at L3-5 except the right L4.

**Fig. 2:**
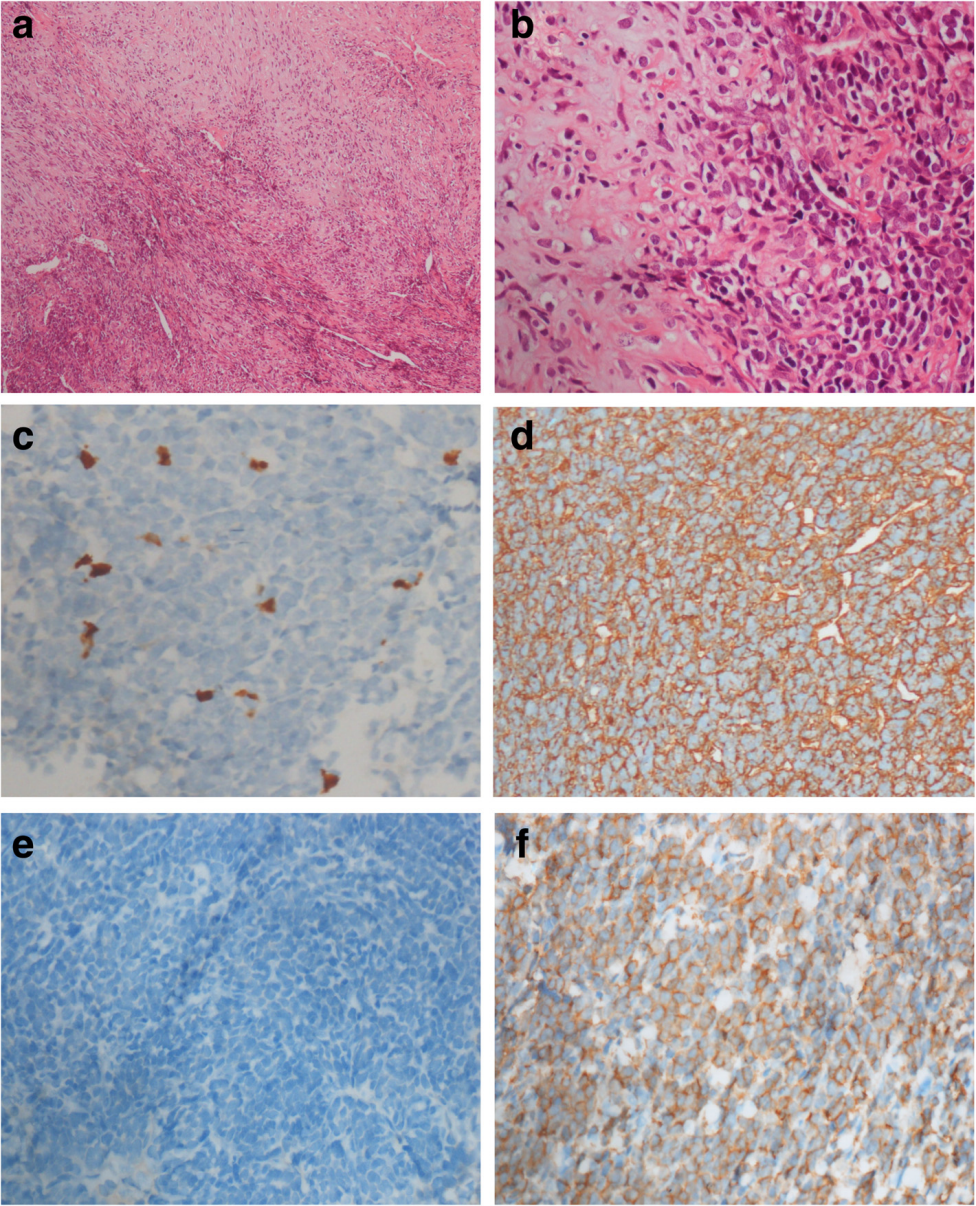
Postoperative histopathological findings. The tumor showed primitive round or spindle-shaped undifferentiated cells alternating with zones of well-differentiated cartilage (**a** HE × 200, **b** HE × 400). **c**–**f** Histopathological examination of the specimen stained positive for S-100, Vimentin, NSE, and CD99, respectively

Microscopic examination of tumor biopsies showed that the tumor was composed of round, oval, or spindle-shaped mesenchymal cells and dispersed cartilage islands. The mesenchymal cells were more or less the same size, with few cytoplasm, dark stained nuclei, rare nucleus division, and abundant blood sinus, indicating localized necrosis. Immunohistochemistry of biomarkers including Vimentin, Bcl-2 and CD99 (mesenchymal cells), CD 34 (vessels), S-100, and NSE (chondroid areas), was used for further diagnosis, whose results were all positive (Fig. 2), while assay for FLI-1 showed negative. Based on these examinations, the tumor was diagnosed with mesenchymal chondrosarcoma.

**Fig. 3:**
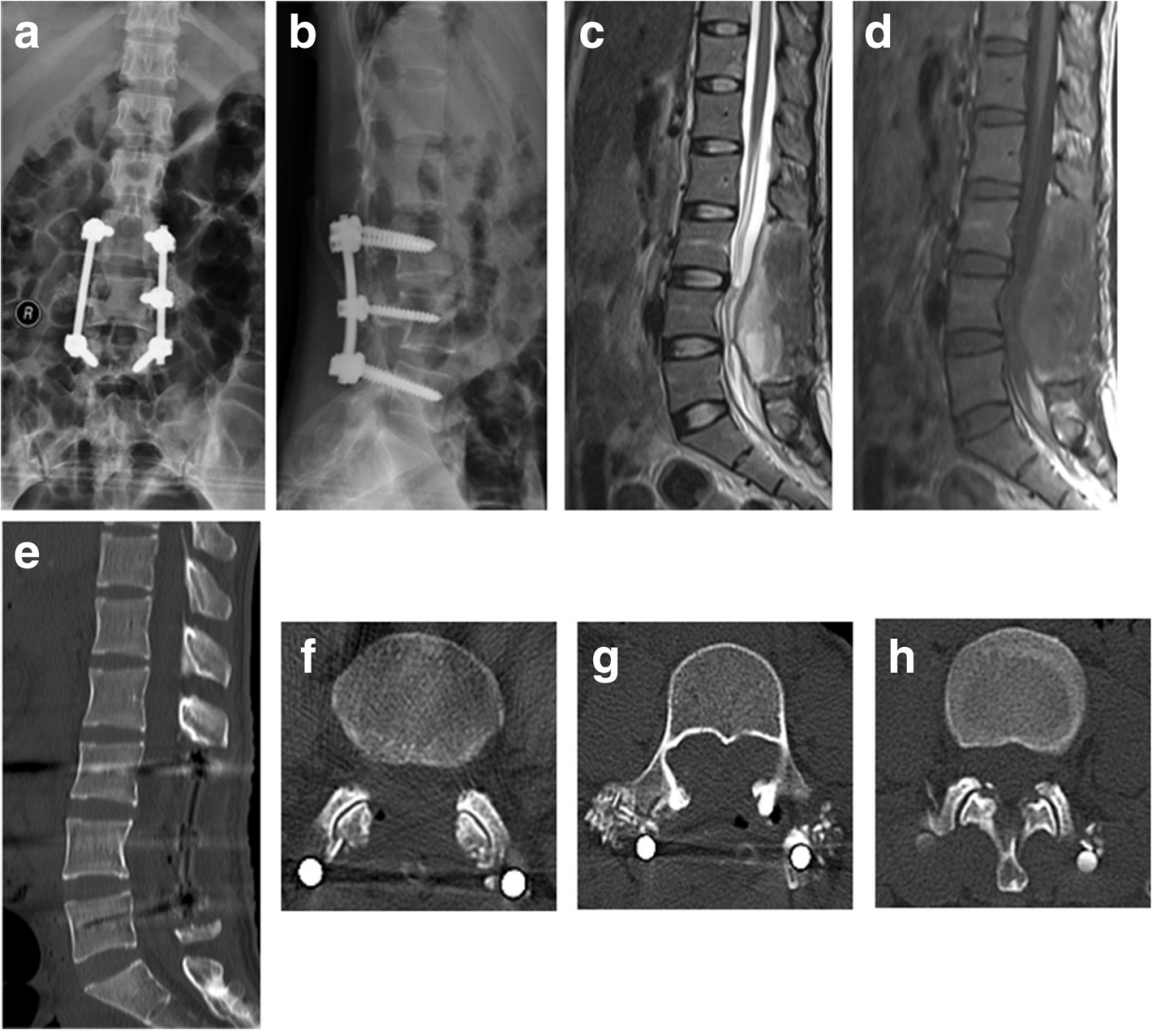
The postoperative X-rays and CT and MRI scans. (**a**-**b**) Images of postoperative X-rays. Sagittal T2WI (**c**) and T1WI (**d**) of the postoperative MRI images show the mass has been completely resected. (**e**-**h**) Images of postoperative CT confirm that the mass has been removed. **e** shows the sagittal plain and **f**-**h** represent the level of L3, L4 and L5, respectively

**Fig. 4:**
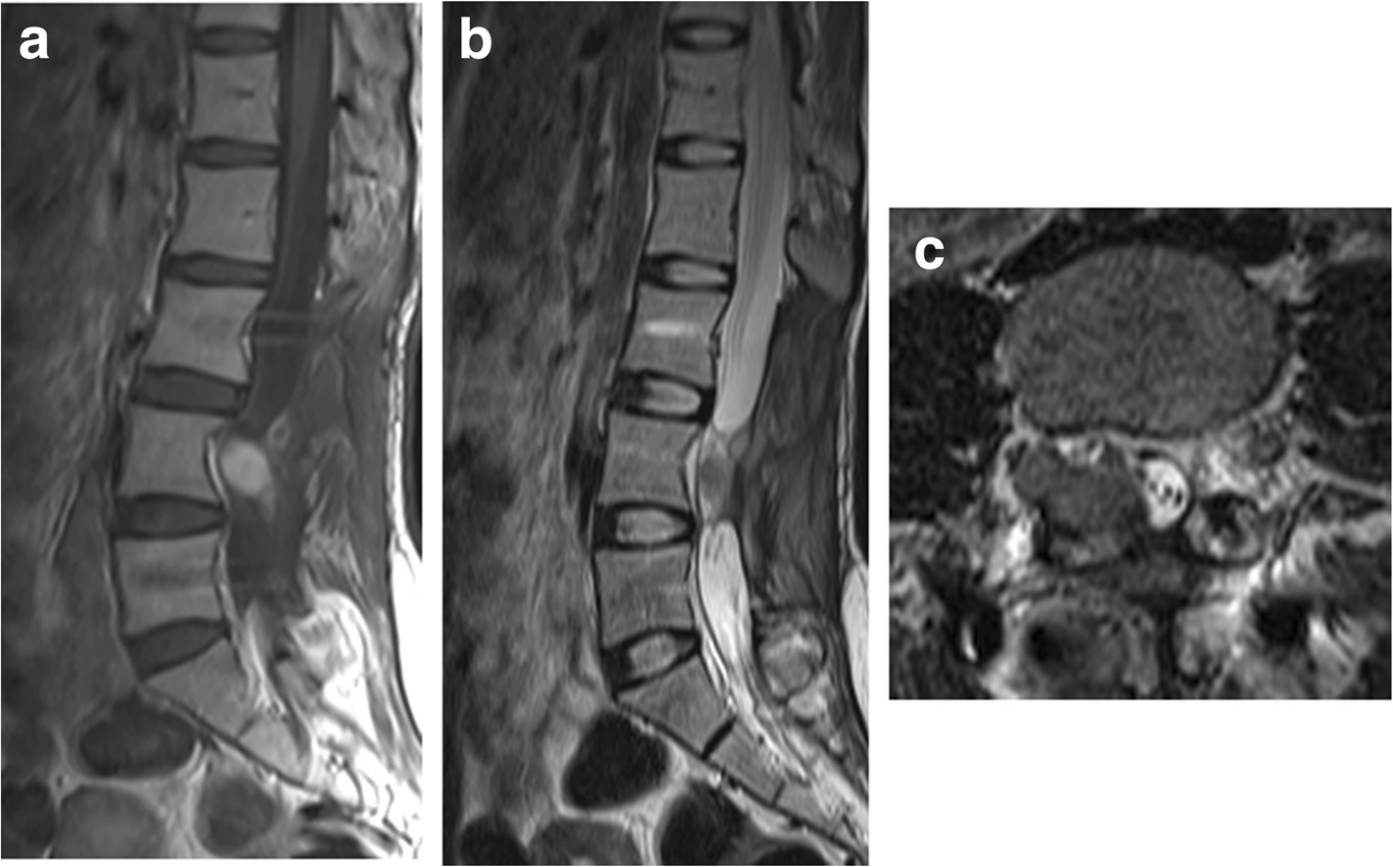
MRI images 5 months after the surgery. Sagittal T1WI (**a**), T2WI (**b**), and axial T2-WI (**c**) find a soft tissue mass at the L4 level of spinal canal, confirming tumor recurrence

The pain went away, and no any neurological deterioration happened after surgery. No further treatment was carried out when the tumor had been completely resected, which had been confirmed by the postoperative CT and MRI (Fig. 3). The patient remained free until she complained of right sciatic pain 5 months later. No dissemination was found upon the chest X-rays, and the internal fixation was still stable. MRI showed a mass in the right side of the spinal canal on the L4 level, suggesting recurrence of the tumor (Fig. 4). The tumor was removed completely under microsurgery once again, after which she felt no pain. Postoperative histopathological examination confirmed tumor recurrence, and MRI re-examination revealed no intraspinal mass. Subsequently, the patient went on radiotherapy, the dose of which was 55.4 Gy/28 F.

### Discussion

Mesenchymal chondrosarcoma (MCS) is a rare malignant chondrosarcoma. It was in 1959 that Lightenstein and Bernstein [1] first described MCS arising from the bone. Later, in 1964, Dowling [2] reported the first case of MCS in non-osseous tissues. Previous literatures announced the incidence of MCS was associated with age (younger) and gender (female) in the past few decades. However, up to now, an increasing number of reports suggest that MCS can occur anywhere in the body and at any age, with a male-to-female ratio of 1:1 [4–8]. The youngest patient was a 1-day neonate [9], and the oldest one was 80 years old [8]. Primary MCS can arise from the bone such as the pelvic, sacral, jaw, femur, humerus, and ilium andoscalcis [5, 10, 11], as well as in extraskeletal locations such as the brain, kidney, spinal dura, and pancreas [12–15].

Here, we report a 26-year-old female patient with MCS in the lumbar spinal canal. Generally, most of the spinal tumors with dumbbell shape are from nervous system, such as schwannomas or meningiomas [16]. To date, we only found six cases of primary intraspinal dumbbell-shaped MCS (Table 1) [17–22]. What rather special in our case is that it is not only the unique case of MCS with a history of 12 years and without metastasis, but also, as far as I know, is the first reported case of intraspinal dumbbell-shaped MCS with massive calcifications.

**Table 1:** Literature review of primary intraspinal dumbbell-shaped mesenchymal chondrosarcomas

Study	Age (years) and gender	Location	Calcification	Treatment	Adjuvant therapy	Outcome
Chan HS et al. 1984 [17]	10/F	T3-4 extradural	−	Total resection	Radiation therapy and chemotherapy	Alive, 18 months follow-up
Reif J et al. 1987 [18]	3	L1–5 intra- and extradural	−	NA	Radiation therapy and chemotherapy	Died of brain metastasis
Di LN et al. 1989 [19]	40	L5–S1 extradural	−	Total resection	Radiation therapy and chemotherapy	Alive, 5 years follow-up
Rushing EJ et al. 1996 [20]	48	T10–12 extradural	−	Total resection	Radiation therapy	Died of brain metastasis
Bae GS et al. 2011 [21]	25/M	T7 intra- and extradural	+	Total resection	Radiation therapy and chemotherapy	Alive, 2 years follow-up
Iida T et al. 2014 [22]	10	T9–10 intra- and extradural	−	Total resection	−	Alive, 3 years follow-up
Current case	26/F	L3-5 extradural	+	Total resection	Radiation therapy	Alive

Pain or swelling of the lesion often leads to the early diagnosis of MCS. However, currently, detection of MCS is often delayed for nonspecific signs and symptoms, unless when the compression of the spinal cord or nerve roots cause initial symptoms of radicular pain, numbness, or muscle weakness. Yet, intraspinal MCS may later have onset of symptoms [23], probably due to the space of the spinal canal. In this case, the patient had restricted ankle movement since she was 14 years old, but it was not until 12 years later that she felt severe radiating pain and came to see doctors, when she already had typical symptoms such as foot drop.

Although imaging examinations like MRI or CT scan, which are already the best choices, can provide some evidence for diagnosis of this tumor [24, 25], there have not been distinct radiologic features that can help to distinguish MCS from other tumors. Few doctors who have tried fluorodeoxyglucose positron emission tomography (FDG-PET) just found hypermetabolism in the lesions matched with enhancement on spine [7, 26]. Radiographs usually give an appearance of soft tissue mass or osteolytic, ill-defined lesion, or well-defined borders with sclerosis. The tumor generally shows a lobulated mass with distinct edges and appears isointense on T1WI and T2WI images, with marked homogeneous enhancement. In some cases, the soft tissue lesion is mixed in different degree of mottled calcifications [27]. T2WI images can clearly show both calcified and noncalcified area, while enhanced MR images reveal the heterogeneous enhancement of both regions [28]. In our case, we can see a lobulated, inhomogeneous density and well-defined mass with a large number of matrix calcifications, showing low-signal intensity on T2WI images in the MRI, which is quite different from the common case with isointense. However, due to the unreliability of imaging examination to identify MCS, doctors should also consider the hemangioblastoma, neurofibroma, and schwannoma while making a diagnosis.

The MCS is a hard or fish-meat like, grayish-white or grayish-red mass, mixed with calcifications sometimes [6, 20]. During operation, we discovered a grayish-red mass with a great quantity of abnormal ossification. Moreover, judging from the damaged L5 nerve root and her onset symptoms of dorsiflexion difficulty 12 years ago, we speculated that the patient had a potentially prolonged course of the tumor. As yet, no metastasis happened. These may be related to its extensive calcifications which may be a sign of slow tumor growth. Studies had presented that calcifications were associated with the prognosis of some tumors. Danse et al. [29] discovered calcifications remaining unchanged in a case of abdominal Burkitt lymphoma in an 8-year-old boy after 4 years of follow-up. Joshi et al. [30] reviewed 211 patients with neuroblastoma and found that calcification was one of the histologic features associated with better prognosis. On the other hand, researchers also found that during radiotherapy and chemotherapy, the primary tumor may be calcified, which would get meaningful clinical benefits and maintain a better quality of life [31, 32]. It seems an empirically established fact that calcifying tumors are likely to be relatively benign and probably hinder the spread of tumor [33]. However, the mechanism of matrix mineralization is still unknown. Matrix vesicles and bone isoenzyme of alkaline phosphatase are the two most correlative factors in the process of calcification from the aspect of cell-biology [34]. It can be seen from X-rays that the posterior edge of L4 were pressed towards central vertebra, which were caused by long-term tumor compression but without invading. Under light microscope, the tumor has a bi-directional differential feature which consists of undifferentiated small-round mesenchymal cells and islands of hyaline cartilage. Immunohistochemistry of biomarkers such as Vimentin, S-100, CK, CD99, NSE, CD56, CD34, and FLI-1 may help us to differentiate some of these tumors, such as hemangiopericytoma and Ewing’s sarcoma.

The therapy and prognosis of MCS still remain controversial, especially for the use of postoperative radiotherapy and chemotherapy [7, 20]. Information of MCS mainly gained from single case reports, small case series, or literature review, and there are only 12 papers providing data more than ten cases [4–8, 10, 20, 35–39], among which eight papers showed the data of 5-year or 10-year survival rate (Table 2). Because of the rarity of MCS, treatment approaches still lack adequate evidences. Despite the fact that differential diagnosis based on MRI findings has no high specificity, it is of great importance for surgical planning. The decision about how to remove the tumor seems to be more important than to assume what the tumor is. There are general agreements on complete surgical resection of the tumor [6], but in some certain positions such as the maxillofacial region, or central nervous system, this may not be applied. Adjuvant therapy can be considered if the tumor is an incomplete resection [40]. Although the effectiveness of additional chemotherapy or radiotherapy was still unclear, they have been tried to be applied in clinical practice [6, 10, 37]. In general, people who support the adjuvant therapy think that radiotherapy is a choice for local control while chemotherapy is a choice for systemic control or metastasis [1, 6]. Radiotherapy is assumed to be nonsensitive to low differentiating tumors; however, MCS, with high differentiation cells, may benefit from it. In 1981, Harwood et al. [37] advanced the opinion that adjuvant therapy should be performed for MCS when necessary, because in other small-round cell malignant tumors, such as Ewing’s sarcoma and osteogenic sarcoma, this therapy is effective. Huvos et al. [5] thought that chemotherapy is better for malignant tumor that originated from small-round cells which similar to Ewing’s sarcoma than that originated from spindle-cell. Researches by Anna Maria Frezza et al. [8] showed that chemotherapy administration in patients with localized disease was associated with fewer recurrences. Satoshi Kawaguchi et al. [41], on the other hand, found that fewer recurrences may owe to radiotherapy. In fact, the effects of both chemotherapy and radiotherapy for MCS are still controversial, for lack of randomized controlled trials and no detailed guidelines. In the present case, adjuvant therapy was judged to be unnecessary initially, considering the complete excision of the tumor with no distant metastases. However, the patient with relapse in situ in a short time, after the second surgery, in case of tumor recurrence and metastasis, the radiotherapy was carried out.

**Table 2:** Literature review of MCS outcomes

Study	Patients	Median overall survival (years)	5-year survival rate (%)	10-year survival rate (%)
Dabska M et al. 1983 [36]	19	1.92	35	20
Huvos AG et al. 1983 [5]	35	3.16	42	28
Nakashima Y et al. 1986 [6]	23	–	54.6	27.3
Vencio EF et al. 1998 [10]	19	–	82	56
Knott PD et al. 2003 [39]	13	11.3	64	55
Cesari M et al. 2007 [35]	26	–	–	21
Dantonello TM et al. 2008 [7]	15	–	–	67
Frezza AM et al. 2015 [8]	113	17	70	54

Prognosis in MCS varies from person to person. Previously, the prognoses of MCS were based on the research of 111 patients of Nakashima et al. [6]. In the retrospective study, they presented a group of 23 patients from the Mayo Clinic, the 5-year and 10-year survival rate were 54.6 and 27.3 %, respectively. In February 2015, Anna Maria Frezza et al. [8] reported 113 patients of MCS from the European Musculoskeletal Oncology Society (EMSOS) and evaluated their prognostic factors and outcomes. The median overall survival was 17 years, and the 5-year and 10-year estimated survival rates were 70 and 54 %, respectively, which were higher than those reported by Nakashima et al. [6]. Patients who presented brain metastasis or lung metastasis as the disease progressed will have a poor prognosis. As for MCS in the intraspinal canal, it may have early symptoms caused by spinal cord or nerve roots compression, which leads to earlier diagnosis compared with MCS in other tissues, thus having a relatively better prognosis. But in general, since MCS has a certain relapse and metastasis rate, we tend to perform complete resection and adjuvant therapy and do regular examinations.

## Conclusions

Mesenchymal chondrosarcoma (MCS) is a very rare tumor, and it is difficult to distinguish it from many other kinds of tumors. When masses were discovered in spinal canal by imagings, we could not identify it merely through its shape, intensity, and other features. MCS, rare as it is, should also be considered. Actually, sometimes, solitary or punctate calcifications could be seen in tumors, which may influence or reflect the growth of tumor and disease progression. In this case, the patient had a long history of drop foot due to the injury of L5 nerve root and no tumor metastasis, which may be related to its calcification. According to imagings, decisions about making appropriate operation plans seem to be more important than to assume what the tumor is. Through CT or MRI, we can clearly see the margin of the tumor and decide how to remove the tumor. Although we performed a total resection of the tumor, she experienced tumor recurrence half a year later. Hence, complete resection, adjuvant therapy, and regular examinations are recommended to perform for MCS patients.

## Abbreviations

CT, computed tomography scan; L5, the fifth lumbar; MCS, mesenchymal chondrosarcoma; MRI, magnetic resonance imaging scans
